# Perspective: Biophysical regulation of cancerous and normal blood cell lineages in hematopoietic malignancies

**DOI:** 10.1063/1.5025689

**Published:** 2018-05-22

**Authors:** Sing Wan Wong, Stephen Lenzini, Jae-Won Shin

**Affiliations:** Department of Pharmacology, College of Medicine, University of Illinois at Chicago, Chicago, Illinois 60612, USA and Department of Bioengineering, College of Medicine, University of Illinois at Chicago, Chicago, Illinois 60612, USA

## Abstract

It is increasingly appreciated that physical forces play important roles in cancer biology, in terms of progression, invasiveness, and drug resistance. Clinical progress in treating hematological malignancy and in developing cancer immunotherapy highlights the role of the hematopoietic system as a key model in devising new therapeutic strategies against cancer. Understanding mechanobiology of the hematopoietic system in the context of cancer will thus yield valuable fundamental insights that can information about novel cancer therapeutics. In this perspective, biophysical insights related to blood cancer are defined and detailed. The interactions with immune cells relevant to immunotherapy against cancer are considered and expounded, followed by speculation of potential regulatory roles of mesenchymal stromal cells (MSCs) in this complex network. Finally, a perspective is presented as to how insights from these complex interactions between matrices, blood cancer cells, immune cells, and MSCs can be leveraged to influence and engineer the treatment of blood cancers in the clinic.

NOMENCLATURE

ALL: Acute lymphoid leukemia
AML: Acute myeloid leukemia
APC: Antigen presenting cell
BCR: B-cell receptor
BM: Bone marrow
CLL: Chronic lymphocytic leukemia
CML: Chronic myeloid leukemia
CXCL12: CXC-chemokine ligand 12
CXCR4: CXC-chemokine receptor type 4
DC: Dendritic cell
E: Young's modulus
GvHD: Graft-versus-host disease
GvT: Graft-versus-tumor
HSC: Hematopoietic stem cell
LepR: Leptin receptor
LOX: Lysyl oxidase
LSC: Leukemia stem cell
MMP: Metalloproteinase
MSC: Mesenchymal stromal cell
NG2: Neuron-glial antigen 2
PD-1: Programmed cell death protein-1
SDF-1: Stromal-derived factor-1
SIRPα: Signal regulatory protein α
TCR: T-cell receptor
VCAM-1: Vascular cell adhesion molecule-1
VEGF: Vascular endothelial growth factor
YAP1: *Yes*-associated protein-1

## I. INTRODUCTION

Significant progress has been made in the biology, diagnostics, and therapeutics of cancer over several decades. More recent progress has unveiled a key insight: cancer needs to be understood by considering its microenvironments since they contribute to initiation, metastatic potential, and drug resistance.^1^ Various genomic and proteomic approaches have elucidated biochemical components, cellular types, and signaling pathways that regulate cancerous microenvironments, followed by extensive validation of individual genes using animal models. By combining these approaches in cancer biology with technologies in bioengineering—most notably, biomaterial design and physical probing methods—it has been increasingly appreciated that biophysical cues play important roles in cancer. Most studies on biophysical regulation of cancer have explored the contribution of solid mechanics, including stiffness (stress versus strain) of the extracellular matrix and fluid mechanics, such as shear stress present in blood and lymphatic flow. Additionally, some of the known genes in cancer, especially those that encode structural proteins such as cytoskeletons, are now beginning to be reinterpreted in the context of biophysical regulation and cancer mechanobiology.

**FIG. 1.**
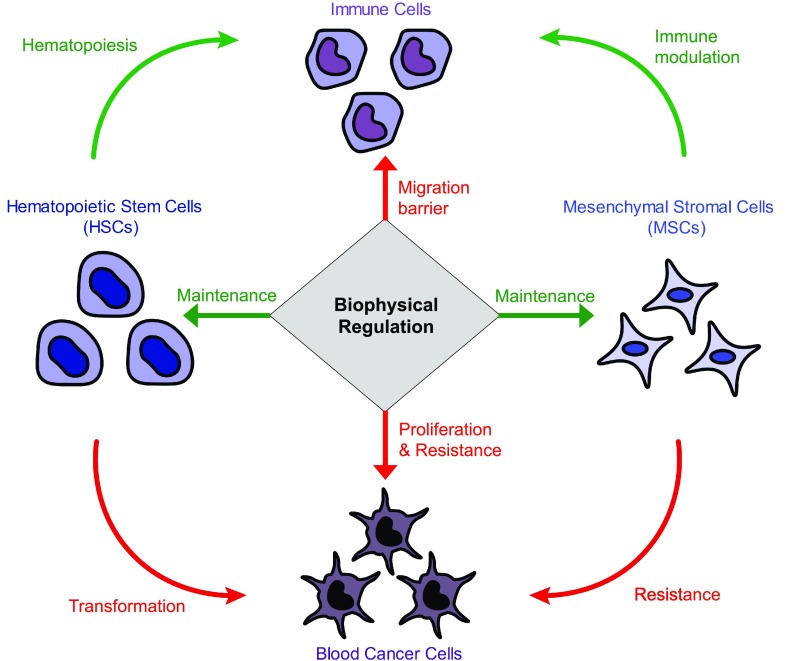
Understanding biophysical regulation of different cellular components in blood cancers. The role of extracellular matrix mechanics is highlighted. Green arrows highlight the functions that may benefit cancer treatment, while red arrows indicate the functions that may promote cancer. Biophysical cues from the matrix are known to play important roles in maintaining HSC functions and directing MSC differentiation. HSCs contribute the turnover of immune cells, while MSCs are known to modulate immune cells. However, the dense matrix represents a barrier for immune cells to migrate through and interact with cancer cells. Biophysical cues from the matrix are also known to regulate proliferation and chemoresistance of cancer cells. Additionally, blood cancer cells are known to originate from HSCs when they are mutated, while they also become chemoresistant when they interact with MSCs.

While previous work has mostly focused on biophysical regulation of cells in solid tumors, it is important to note that substantial clinical progress has been made mostly with blood cancers, including chemotherapy and hematopoietic stem cell (HSC) transplantation. Recent clinical success in immunotherapy demonstrates that immune cells—blood cell lineages—can be targeted to treat both blood^2^ and solid cancers.^3^ Both blood cancer cells and immune cells interface with the extracellular matrix and non-hematopoietic cells, especially stromal cells, in microenvironments.^1^ Therefore, understanding how biophysical cues from the matrix regulate blood cancer cells and immune cells both directly and indirectly via stromal cells can potentially serve as an important platform that may provide information about treatment strategies for a broad range of cancer (Fig. 1). In this perspective, we will first summarize key knowledge related to hematopoiesis, hematopoietic malignancies, leukemia stem cells (LSCs), and hematopoietic stem cell niches, followed by a description of how biophysical cues from the matrix may impact the pathophysiology and pharmacology of blood cancer cells. We will then discuss how understanding biophysical regulation of immune cells in cancer microenvironments may provide novel insights for immunotherapy. We will speculate how physical forces may impact blood cancer cells, blood cell turnover, and immunity in cancer by regulating stromal cell functions. Finally, we will discuss how insights into mechanobiology might be translated to the clinical setting for the treatment of cancer.

## II. HEMATOPOIESIS, HEMATOPOIETIC MALIGNANCIES, AND LEUKEMIA STEM CELLS

**FIG. 2.**
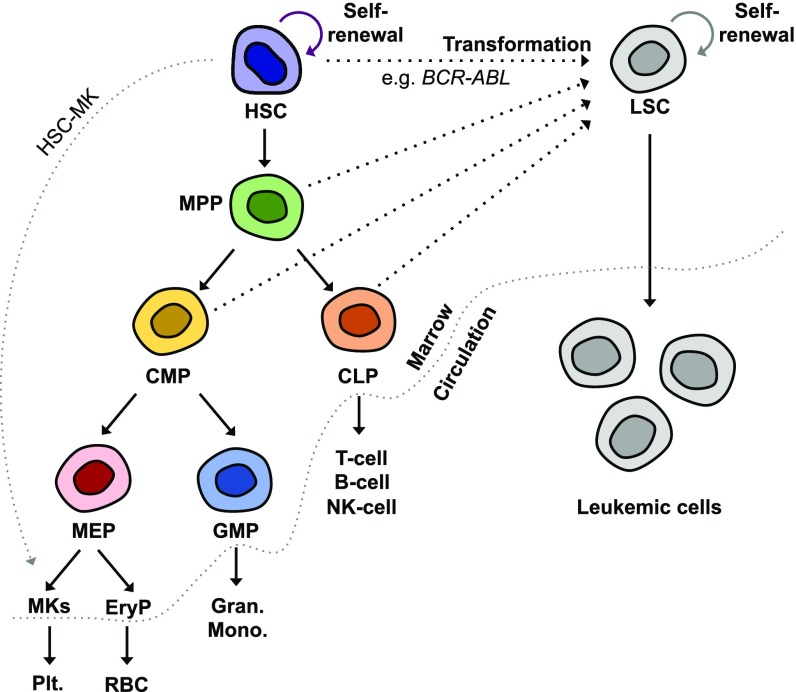
Hierarchical organization of normal hematopoiesis and leukemic transformation. A conventional model of normal hematopoiesis is shown on the left where different blood lineages are derived from hematopoietic stem cells (HSCs). HSCs give rise to multipotent progenitors (MPPs), which lose self-renewal capability. MPPs differentiate into common myeloid progenitors (CMPs) and common lymphoid progenitors (CLPs). CLPs produce lymphoid cells [T-cells, B-cells, and Natural Killer (NK)-cells]. CMPs further differentiate into megakaryocyte-erythroid progenitors (MEPs) and granulocyte-monocyte progenitors (GMPs). GMPs produce granulocytes (gran.) and monocytes (mono.), while MEPs generate megakaryocytes (MKs) and erythroid progenitors (EryPs). Fragmentation of mature MKs under shear stress makes platelets, while nucleation of EryPs leads to red blood cells (RBCs). Terminally differentiated cells subsequently egress the marrow and are distributed throughout different organs. A recent example is highlighted where a newly discovered subset of HSCs is exclusively differentiated into the MK lineage (HSC-MK).^5^ Leukemia stem cells (LSCs) are derived from the oncogenic transformation of HSCs. However, the transformation of progenitors can also turn them into LSCs depending on oncogenic mutations that define leukemia subtypes. An example is shown where *BCR-ABL* transforms HSCs but not progenitors to generate LSCs in CML.^17^

The hematopoietic system has served as a model to understand how stem cells give rise to different lineages and how this process is perturbed in malignancies. A combination of fluorescence activated cell sorting and functional assays such as the colony-forming unit assay and transplantation has revealed a hierarchical system of blood cell lineages emanating from stem cells (Fig. 2), which can be separated based on surface receptor expression. This map has been extended to human cells through xenotransplantation in genetically engineered, immunocompromised mouse models.^4^ In general, HSCs can be distinguished from progenitors based on long-term (≥4 months), multilineage engraftment after transplantation and successful engraftment after serial transplantation into a new host. Progenitors can be detected based on their ability to form colonies from individual cells. Overall, Hematopoietic lineages are classified into myeloid and lymphoid. Myeloid lineages are further classified into two groups—(1) Erythroid lineages that lose nuclei, eventually giving rise to red blood cells, and megakaryocyte lineages that produce platelets; (2) Lineages that retain nuclei, including monocytes, granulocytes, and dendritic cells (DCs). Lymphoid lineages primarily include B-cells, T-cells, and natural killer (NK) cells. While the accepted foundation of hematopoietic hierarchy holds, the lineage map is continuously revised to show novel subpopulations and lineage bias of some HSCs, such as those that directly differentiate into the megakaryocyte lineage,^5^ reflecting a level of heterogeneity in hematopoiesis.^6^

Hematopoietic malignancies are classified based on the organ where cancerous cells are located (marrow and blood for leukemia and lymph nodes for lymphoma), the differentiation status of abnormal cells (more primitive cells for acute and more mature cells for chronic), and the affected lineages (myeloid and lymphoid). Chronic malignancies that affect myeloid lineages are broadly termed chronic myeloproliferative neoplasms (CMNs). CMNs are further classified into chronic myeloid leukemia (CML) that shows genetic translocation in chromosome 22 (“Philadelphia chromosome” with a *BCR-ABL* fusion gene) and the Philadelphia-chromosome negative disorders, including essential thrombocythemia, polycythemia vera, and primary myelofibrosis.^7^ Acute myeloid leukemia (AML) is characterized by rapid proliferation of immature myeloblasts and is associated with a number of genetic mutations, most notably those of the mixed lineage leukemia (*MLL*) gene.^8^ Chronic lymphocytic leukemia (CLL) occurs in B-cells and is the most common leukemia subtype in adults.^9^ Multiple myeloma is a malignancy of terminally differentiated, plasma cells, which are a subset of the B-cell lineage.^10^ Acute lymphocytic leukemia (ALL) also develops mostly from the B-cell lineage, but 25% of cases develop from the T-cell lineage.^11^ Lymphomas can be classified into two forms. Hodgkin's lymphoma accounts for 10% of all lymphomas and is diagnosed based on the presence of giant multinucleated cells called Reed-Sternberg cells.^12^ In contrast, non-Hodgkin's lymphoma shows a diverse spectrum of subtypes depending on morphology, genetics, and surface marker phenotyping.^13^

Hematopoietic malignancies are now understood in the context of the hematopoietic hierarchy, especially for leukemias (Fig. 2). Efforts over the past two decades have established the concept of leukemia stem cells (LSCs) in which leukemias originate from a minority of malignant cells that possess stem cell-like functions, including long-term repopulation and self-renewal.^14^ Existence of human LSCs was first shown by transplanting purified marrow cells from AML patients into immunocompromised mice and demonstrating that only the primitive HSC (CD34^+^CD38^−^) population can cause AML in mice.^15^ Interestingly, subsequent studies show that the overexpression of AML-causing *MLL* mutants can transform not only primitive HSCs but also myeloid progenitors that lack self-renewal capability.^16^ In contrast, the overexpression of CML-causing *BCR-ABL* modifies HSCs that possess inherent self-renewal capacity, but it does not modify progenitor cells.^17^ While transplant of purified HSCs but not progenitors recapitulates CLL in xenograft mice,^18^ different subpopulations have been shown to possess the leukemia-initiating property in ALL.^19^ In sum, these findings highlight that LSCs primarily originate from HSCs, but some LSCs can also be derived from more differentiated progenitors depending on the leukemia subtype.

## III. BONE MARROW MICROENVIRONMENTS: BIOMECHANICAL PERSPECTIVE

**FIG. 3.**
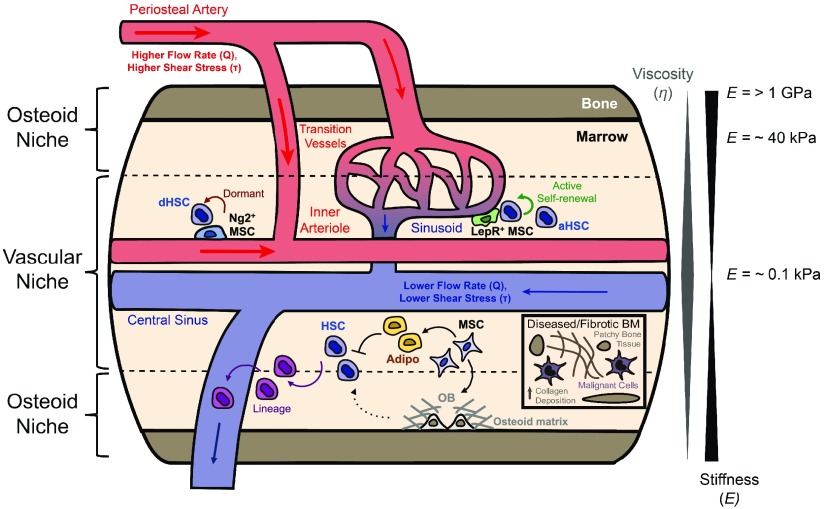
A schematic of the bone marrow microenvironment with key stromal cell components and biomechanical characteristics. Tissue becomes softer and fluids more viscous when moving inward radially from the surface of the periosteum. Periosteal arteries lining the surface of the periosteum impose high flow rates and shear stresses that decrease as blood moves through transition vessels followed by sinusoids and eventually the central sinus, leading to systemic venous circulation. In marrow, the osteoid and vascular niches promote different cellular phenotypes based on their mechanical attributes. Hematopoietic stem cells (HSCs) are primarily located near sinusoidal vasculature. Mesenchymal stromal cells (MSCs) positive for leptin receptor (LepR^+^) are located at sinusoids and promote active self-renewal of active HSCs (aHSCs), while MSCs positive for neuron-glial antigen 2 (Ng2^+^) near arterioles support HSC dormancy (dHSCs). HSCs differentiate into hematopoietic lineages, which eventually exit the marrow through the central sinus. MSCs can differentiate into adipocytes (Adipo), which limit HSC proliferation, and osteoblasts (OB), which help to maintain HSCs. Diseased and/or fibrotic regions of marrow, such as in primary myelofibrosis, show increased bone formation and enhanced deposition of collagen and infiltration of malignant cells (inset).

The bone marrow (BM) is the primary organ that maintains HSCs and supports hematopoiesis in adults. It is important to highlight that the BM consists of an incredible diversity of biomechanical cues (Fig. 3). In general, the inner marrow is softer (*E* = 0.2–25 kPa)^20,21^ than the osteoid matrix (*E* = 40–100 kPa) deposited by osteoblasts lining the inner bone surface.^22^ Detailed analyses of the BM *in situ* by atomic force microscopy (AFM) at the microscale confirm that the marrow is generally soft (*E* = 0.1 kPa), while the regions closer to the inner bone surface show different stiffness values ranging from *E* = 2–100 kPa.^23^ It is also important to consider fluid mechanics in the BM. Blood flows into the BM through periosteal arteries lining the periosteum and penetrates through the endosteum to form interior arterioles. Sinusoidal circulation begins with transition vessels stemming from arteriolar circulation to the sinusoids, an extensive venous network distributed throughout the marrow. Thus, transition vessels and sinusoids represent the circulatory regions where blood flow transitions from the high to low flow rate and shear stress. Sinusoidal circulation consolidates into the central sinus, which eventually connects to systemic venous circulation. While the overall marrow viscosity has been reported to be ∼100 mPa^ ^s, the central marrow regions are more viscous than other regions because of fluid transport and exchange through large central vessels.^21,24^ Together, these studies indicate that the outer regions of the BM may act more like elastic solids, while the central regions act more like viscoelastic solids.

*In vivo* studies have revealed cellular components in the BM that are required to maintain HSC functions.^25,26^ Recent studies show that most HSCs are primarily localized in the vascular niche near sinusoids and the central sinus, while some can be identified near arterioles.^27^ By using conditional depletion of cells *in situ*, it was shown that leptin receptor positive (LepR)^+^ mesenchymal stromal cells (MSCs) localized on sinusoids,^28^ and neuron-glial antigen 2 positive (NG2)^+^ MSCs localized on arterioles^29^ are required for the maintenance of HSCs in the BM. Since HSCs are abundant near sinusoids, LepR^+^ MSCs likely support self-renewing active HSCs, while NG2^+^ MSCs likely support slowly cycling, dormant HSCs.^30^ Like conventional MSCs, LepR^+^ MSCs are capable of differentiating into osteogenic and adipogenic lineages.^31^ Previous imaging and functional studies show that osteolineages likely maintain HSCs indirectly and play more direct roles in regulating progenitors.^27^ In contrast, adipocytes are known to limit the number of HSCs *in vivo.*^32^ These results highlight the importance of cellular components from MSC lineages in regulating HSC functions.

Emerging studies show the contribution of biomechanical cues in regulating the number of functional HSCs. In embryonic hematopoiesis, HSCs originate from endothelial cells that experience shear stress. Interestingly, fluid shear increases the number of embryonic HSCs^33^ by activating prostaglandin E_2_.^34^ Whether this insight applies to adult hematopoiesis remains unclear. While the direct contribution of matrix proteins secreted by specific cell types in HSC maintenance remains to be observed *in vivo*, high levels of fibrillar collagen and fibronectin are localized at the endosteal surface, whereas basement membrane proteins are localized near the vasculature.^35^ Recent studies show that soft (*E* = 0.3 kPa) substrates maintain HSCs when they are functionalized with tropoelastin or fibronectin,^36,37^ while stiff (*E* = ∼40 kPa) fibronectin functionalized substrates increase the proliferation of multipotent progenitors.^38^ Taken together, these findings suggest biomechanical influences in the regulation of hematopoiesis.

## IV. BIOPHYSICAL INTERACTIONS BETWEEN MALIGNANT BLOOD CELLS AND THE EXTRACELLULAR MATRIX

How solid mechanical cues from the extracellular matrix impact the biology of hematopoietic malignancies is now being studied by coupling biomaterial strategies with molecular biology. With a number of subtypes documented based on genetic mutations, hematopoietic malignancies can potentially serve as an important system to understand complex relationships between cancer genotypes and their sensitivity to biophysical cues.

Malignant blood cells express several molecular components required to sense biophysical cues presented by the matrix. On the cell membrane, some integrin receptors, most notably α4^39^ and β3,^40^ are known to be required for leukemia cell growth and drug resistance. While hematopoietic lineages in general show less obvious focal adhesion kinase clustering compared to non-hematopoietic cells upon adhesion, it is upregulated in some AML cells and is associated with higher cell motility and drug resistance.^41^ The A isoform of Myosin-II, which is a principal motor protein in more differentiated hematopoietic cells,^37^ has been shown to be required for leukemia cell engraftment by regulating transmigration.^42^ Small GTPases regulate cytoskeletal rearrangements and are also known to play important roles in leukemia. In CML harboring a *BCR-ABL* mutation, Rac becomes highly active in HSCs.^43^ Cdc42 is shown to regulate asymmetric division of AML cells and to be required for leukemia progression.^44^ Mutations in RhoA are shown to be common in adult T-cell leukemia/lymphoma and contribute to its pathogenesis.^45^ In addition, nuclear components of mechanotransduction regulate leukemia. For instance, while different leukemia cell lines express various levels of intermediate filaments lamin A and C,^46^ their levels are generally low in granulocyte, monocyte, and lymphoid lineages relative to lamin B.^47^ Recent evidence suggests that lamin B1 expression correlates with overall survival in CLL as it is required to limit somatic hypermutations in B cells.^48^ *Yes*-associated protein-1, a mechanosensitive transcription factor,^49^ is known to initiate apoptosis in leukemia cells harboring DNA damage.^50^ Transcriptional coactivator megakaryoblastic leukemia 1 binds to serum response factor, another well-known mechanosensitive transcription factor,^51^ and activates target genes that may contribute to leukemogenesis.^52^ It remains unclear though how these components participate in the mechanosensing of blood cancer cells.

Solid tumors are often diagnosed by physical palpation, and some studies show that stiff substrates facilitate the malignant phenotype, especially by promoting integrin clustering and focal adhesion leading to enhanced cancer cell invasiveness.^53–55^ Since a number of components relevant to mechanotransduction appear to be upregulated in blood cancer cells, this could lead to a speculation that stiff substrates also facilitate malignancy of blood cancers. However, generalizing the concept in mechanosensing of solid tumors to blood cancers is confounded by a number of factors. First, some studies show that rigidification of tumor environments generally occurs at the periphery, while the core region of tumors actually becomes softer.^56^ Other previous studies demonstrate that a soft fibrin hydrogel actually increases the tumorigenic potential of cancer cells, including those from ALL, compared to a stiffer counterpart.^57,58^ How stiffness heterogeneity in tumor microenvironments is relevant to hematological malignancy remains unknown and will need to be tested directly. Second, the intrinsic mechanosensing machinery may be wired differently for hematopoietic cells in comparison to other cell types. For instance, hematopoietic cells generally have larger nuclear to cytoplasmic volume ratios than non-hematopoietic cells, which can potentially impact the distribution of mechanosensitive factors in response to biophysical cues. In addition, lamin-A/C expression—which scales with tissue stiffness^59^—is generally low in differentiated myeloid and lymphoid cells,^47^ and this might influence their ability to relay external biophysical cues to the nuclei. Third, many disease-causing mutations have been reported in blood cancers,^60^ and some of them can potentially interact with the mechanosensing machinery.

Mutations in HSCs are sufficient to cause blood cancer in some healthy recipients, but the disease outcome is also determined by the microenvironments of the recipients.^61^ It will be thus important to elucidate how biophysical cues of the physiological BM may initially regulate genetically transformed malignant blood cancer cells. A recent study demonstrates that some AML cells, including those harboring a *MLL-AF9* mutation, show a biphasic growth pattern as a function of matrix stiffness due to an autocrine inhibitory mechanism.^62^ The biphasic growth as a function of matrix stiffness has also been observed in some lymphoma cells.^63^ Interestingly, this kind of growth pattern is reminescent of early normal hematopoiesis where dormant HSCs rarely proliferate, while active self-renewing HSCs are found near the softer perivascular niche, and differentiated blood cells no longer undergo active proliferation as they exit the marrow into the blood.^64^ Whether this observation is applicable to malignant hematopoiesis as a function of matrix stiffness remains to be investigated.

Effects of matrix stiffness on drug resistance of cancer cells are becoming increasingly understood. While some chemotherapeutic drugs were originally designed to block rapid proliferation of cancer cells, increasing evidence suggests that drug sensitivity may not be a function of cell proliferation in a number of cancers.^65^ This was demonstrated earlier in the context of some solid tumors where cells proliferate faster on stiffer substrates but also show increased drug resistance.^66,67^ In myeloid leukemia cells, there was also no general correlation between the cell proliferation rate and drug potency as a function of matrix stiffness.^62^ Whether matrix stiffness regulates chemosensitivity appears to depend on molecular targets of drugs and mutations that define leukemia subtypes. For instance, *BCR-ABL* and *MLL-AF9* leukemia cells in softer matrices (*E* ≤ 0.1 kPa) show increased resistance to a number of drugs compared to stiffer (*E* = 0.3–3 kPa) matrices, including the drugs imatinib and cytarabine, which are used in the clinic.^62^ However, the same study shows that regardless of matrix mechanics, drugs against the protein kinase B pathway suppress proliferation of *MLL-AF9* cells, while those against the rapidly accelerated fibrosarcoma pathway suppress that of *BCR-ABL* cells. Interestingly, these oncogenes play an active role in decoupling drug sensitivity from matrix stiffness within specific signaling pathways. Therefore, generalizing the principle of cancer “mechanopharmacology” requires a systematic understanding of how mutations affect mechanosensing in cancer cells, which can be facilitated by incorporating hydrogels with tunable mechanics into a high-throughput drug screening system.

As disease progresses, some chronic blood cancer patients show profound physical changes in the marrow (Fig. 3). In particular, patients with primary myelofibrosis, a type of myeloproliferative neoplasm, are known to exhibit extensive deposition of collagen fibrils. While the stiffness of the fibrotic BM has not been formally measured, previous work on other organ systems shows that fibrosis increases the stiffness of normal tissues about an order of magnitude.^68^ At the later stages of BM fibrosis, some areas of BM turn into bone tissue. These overt changes in the BM microenvironment likely limit blood formation in marrow as indicated by patchy hematopoiesis, and more blood cells are produced in other organs, such as the spleen.^69^ Lysyl oxidase (LOX) is known to stabilize collagen fibrils by covalent crosslinking^70^ and contributes to solid tumor progression by matrix stiffening.^53^ Interestingly, megakaryocytes derived from primary myelofibrosis patients show upregulated LOX expression, thereby facilitating collagen crosslinking.^71^ While direct effects of matrix mechanics on the progression of primary myelofibrosis remain unknown, biomaterial strategies, such as minimal matrix models of scars^68^ and interpenetrating polymer networks,^54^ can potentially be used to recapitulate key matrix compositions of the pathological marrow and to vary substrate stiffness independently.

## V. BIOPHYSICAL REGULATION OF IMMUNE CELLS WITH IMPLICATIONS IN CANCER IMMUNOTHERAPY

Engineered chimeric antigen receptor-T cells are now clinically used to treat some leukemias, including ALL, which were previously incurable by chemotherapy or bone marrow transplantation alone.^2^ Inhibitors against immune checkpoint proteins re-activate dormant immune cells so that they can physically interact with cancer cells to kill them.^3^ Cell-based immunotherapies require engineered immune cells to physically infiltrate microenvironments and reach tumor cells after injection. Additionally, checkpoint inhibitors must maintain or restore physical forces required for immune cells to form immunological synapses with antigen presenting cells (APCs) or tumor cells. Both adoptive cell and checkpoint inhibitor immunotherapies need to overcome resident immune cells that may be primed to promote tumor progression.

In general, immune cells possess the ability to physically deform and squeeze through small pores in the matrix or cellular junctions, primarily because low expression of lamin-A makes their nuclei more compliant.^47^ Increased stromal stiffening likely disrupts junctional integrity of endothelial cells,^72^ and this can potentially facilitate the extravasation of molecules and cells.^72,73^ However, tumor microenvironments increase interstitial pressure during growth, and it is known that therapeutic nanoparticles or even antibodies do not readily enter tumors unless collagen production in the stroma is reduced.^74^ Immune cells can migrate through pores as small as ∼3 *μ*m in diameter without requiring matrix degradation by metalloproteinases (MMPs).^75^ Although immune cells are significantly deformable, their nuclear envelope may rupture during deformation; consequently, the envelope must be repaired immediately to ensure cell survival.^76^ Therefore, it is likely that adoptively transferred immune cells require matrix remodeling to initially enter tumors from circulation. T-cells that are adherent to vascular cell adhesion molecule 1 (VCAM-1) are shown to upregulate MMP-2 (gelatinase) and MMP-14 (membrane form) via α4 integrin, which may form molecular complexes during transmigration to degrade the endothelial basement membrane.^77^ Whether changes in tumor stiffness influence T-cells to degrade matrices remains unclear, but a recent study shows that other cell types such as fibroblasts downregulate MMPs on stiff matrices.^78^ Therefore, physical barriers to enter microenvironments of primary myelofibrosis or solid tumors can potentially impair sufficient infiltration of engineered T-cells. Incorporating explicit strategies to improve the entry of engineered immune cells into tumors will likely facilitate clinical translation of cell-based immunotherapy against chronic blood cancers and solid tumors.

For adaptive immune cells, it is clear that mechanical force plays an active role in regulating cell-cell interactions at immunological synapses.^79^ External force is known to directly activate the T-cell receptor (TCR)^80^ and the B-cell receptor (BCR).^81^ A study with dual micropipette aspiration and a biomembrane force probe shows that force promotes catch-bond formation between the TCR and the peptide major histocompatibility complex (pMHC) on APCs in order for T-cells to discriminate between high-affinity and low-affinity antigens.^82^ Actin cytoskeleton assembly and myosin-II-mediated contractility are known to be crucial for mechanosensing and mechanotransduction at immunological synapses.^81,83^ Interestingly, previous studies show that increasing substrate stiffness enhances the activation of T-cells^84,85^ and B-cells.^86,87^ These studies raise the possibility that immune cells in soft tissues, such as the softer core region of tumors, may be activated too weakly to initiate immune responses against cancer cells.

The role of biophysical cues in regulating innate immune cells is beginning to be elucidated, especially for macrophages. Tumor microenvironments can potentially commandeer macrophage functions either by inhibiting phagocytosis or by polarizing them into a protective or “M2-like” phenotype. Although matrix stiffening increases cortical tension within macrophages, which initially enhances phagocytosis,^88^ the concurrent engagement of signal regulatory protein α (SIRPα) in macrophages to CD47 in target cells can deactivate myosin-II activity, thereby eventually inhibiting phagocytosis.^89^ Furthermore, a recent study shows that matrix stiffness increases cell surface expression of SIRPα in macrophages and that marrow-derived macrophages with SIRPα inhibition can effectively clear human tumor xenografts.^90^ Tumor-associated macrophages not only are less phagocytic but also secrete soluble factors that can suppress lymphocyte functions.^91^ Interestingly, forced elongation of macrophages by micropatterning leads to M2 polarization *in vitro.*^92^ Therefore, macrophages can potentially evolve into an M2-like phenotype during migration where they must squeeze through the dense matrix in the tumor stroma. After upregulation of SIRPα, lamin-A, and M2 markers as a result of mechanotransduction in the stiff matrix,^90^ macrophages may become less motile and start contributing to cancer-promoting phenotypes.

## VI. BIOPHYSICAL REGULATION OF STROMAL CELLS TO CONTROL FUNCTIONS OF NEIGHBORING CELLS

Extensive work shows that stromal cells interface with malignant hematopoietic cells to influence drug resistance of malignant hematopoietic cells^93^ and with normal hematopoietic cells to regulate hematopoiesis^25^ and immunity.^94^ While mechanosensing of stromal cells has been studied extensively in the context of their differentiation and migration,^95,96^ its significance in the context of regulating functions of normal and malignant hematopoietic cells remains largely unknown. However, emerging evidence suggests that physical forces regulate the ability of stromal cells to influence their neighboring cells by controlling tissue architecture, remodeling matrices, and secreting paracrine factors.

Lymph nodes become swollen during immune response to accommodate the migration of dendritic cells (DCs) so that they can present antigens to T-cells for activation. Interestingly, a recent study shows that DCs inhibit contractile forces in stroma-derived fibroblastic reticular cells by binding to podoplanin.^97^ This process facilitates the expansion of lymph nodes, liberating T-cells to facilitate their interaction with DCs. This finding highlights an important role of the contractile forces generated by stromal cells in organizing the architecture of lymph nodes and subsequently in controlling the adaptive immune response.

Stromal cells can also impact neighboring cells by generating contractile forces to remodel matrices. On stiff substrates, MSCs increase traction force during cell spreading and secretion of matrix proteins through myosin-II.^59,98^ The deposition of the extracellular matrix by stromal cells has been proposed as a key mechanism that promotes chemoresistance of AML cells.^39^ A recent study shows that priming tumor tissues with Fasudil, an inhibitor of Rho-associated protein kinase (ROCK) used in the clinic, impairs the ability of stromal cells to remodel matrices, decreases cancer cell invasion, and increases sensitivity to conventional chemotherapy.^99^ Coincidentally, an earlier study shows that reversine, a myosin-II inhibitor,^100^ is effective against multiple myeloma cells in the presence but not in the absence of stromal cells.^101^ The studies collectively suggest that contractile forces generated by stromal cells can maintain chemoresistance in cancer by matrix remodeling.

Stromal cells can also regulate neighboring cells in a paracrine manner. A recent study shows that vessel wall shear stress promotes induction of cyclooxygenase-2 in MSCs to suppress activated immune cells.^102^ Effects of matrix mechanics on the secretion of vascular endothelial growth factor (VEGF) from stromal cells have been previously demonstrated.^103–105^ This insight is potentially relevant to understanding the regulation of hematopoiesis as VEGF regulates HSC survival.^106^ In addition, stromal cells can deform matrices by generating contractile forces and release transforming growth factor-β1 (TGF-β1) deposited in the matrix;^107^ interestingly, TGF-β1 is known to facilitate quiescence of HSCs.^108^ The results thus suggest that matrix stiffness regulates the ability of MSCs to secrete paracrine factors that have been previously implicated in maintaining HSCs.

## VII. SCIENTIFIC FUTURE DIRECTIONS: THE ROLE OF MATRIX MECHANICS IN INTERCELLULAR FORCES

**FIG. 4.**
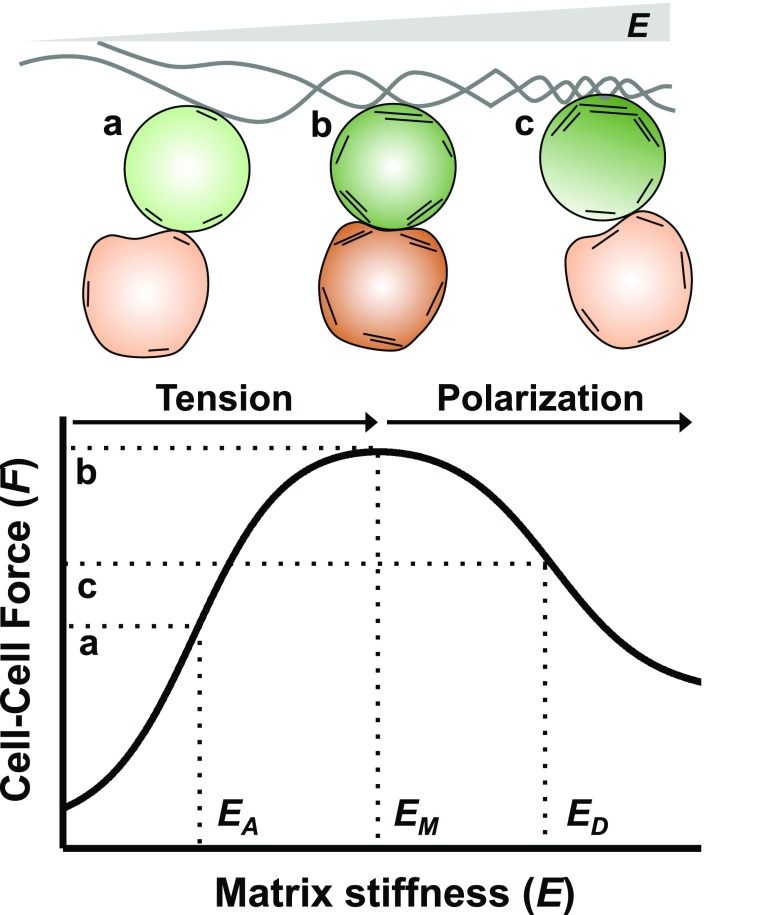
Effects of matrix stiffness on intercellular forces. The cell (green) adhering to the soft matrix may experience low cortical tension and hence interact weakly with the other cell (orange). As the matrix stiffness increases, cortical tension increases, thereby promoting cell-cell interaction. The sensitivity of this process is described by the parameter *E_A_*, the matrix stiffness in which the cell-cell interaction force is half-maximal (a). When matrix stiffness increases beyond the maximal point (*E_M_*), total cortical tension in the cell adhering the matrix may become saturated (b). However, cortical tension may become polarized towards the side where the cell adheres to the matrix. This process can competitively decrease cortical tension on the other side and decrease the cell-cell interaction force to a certain level with the half-maximal stiffness, *E_D_* (c). This leads to a biphasic relationship between cell-cell and cell-matrix interaction forces. This model may be generalizable where E_A_, E_M_, and E_D_ depend on interacting cell types and the matrix. This model can potentially serve as a basic unit to quantitatively understand more complex interactions that involve multiple cells and matrix components.

The role of matrix mechanics in regulating intercellular forces was previously studied with epithelial cells^109^ and endothelial cells^72,73^ but remains generally unknown in the context of hematopoiesis, immunity, and hematopoietic malignancies. A framework can be envisioned to evaluate intercellular forces as a function of matrix stiffness (Fig. 4). The cortical membrane tension of a cell adhering to the matrix is initially increased as matrix stiffness increases.^98^ A cell with higher cortical tension shows larger binding force between surface receptors and their ligands as shown in T-cells.^82^ As the matrix stiffens further, however, some cytoskeletal proteins can become polarized towards the side where the cell interacts with the matrix, as demonstrated in MSCs^110^ and HSCs.^37^ Subsequently, cortical tension is decreased on the side of the cell that interacts with another cell. These observations raise a possibility that intercellular forces show a biphasic pattern as a function of matrix stiffness, depending on whether cells can polarize contractile forces. This notion is supported by an observation that increasing stiffness beyond *E* = 100 kPa starts to decrease T-cell activation.^84^ This framework may help to provide information about an optimal substrate stiffness that can maximize the interaction force between MSCs and HSCs via specific ligand-receptor pairs, such as stem cell factor and CD117, to facilitate the expansion of cord blood cells for HSC transplantation. Since a subset of MSCs (Interleukin-7^+^VCAM-1^+^) in the BM maintains memory CD4^+^ T-cells,^111^ it will also be interesting to determine an optimal matrix stiffness where the maintenance of functional memory T-cells is maximized. Importantly, this framework can be experimentally confirmed by combining dual micropipette aspiration analysis and biomaterial strategies to fabricate microscale substrates that can interact with cells at the single cell level, while intercellular forces can be measured simultaneously.

## VIII. TRANSLATIONAL FUTURE DIRECTIONS: MECHANOTHERAPEUTICS OF HEMATOLOGICAL MALIGNANCIES

Stromal cells are promising novel targets for blood cancer treatment^112^ since they can potentially be modulated to control multiple cell types involved in cancer progression in an integrated manner. Granulocyte-colony stimulating factor has already been used in the clinic to mobilize HSCs from the BM into blood circulation by decreasing stromal-derived factor-1 (SDF-1)^113^ so that HSCs can be readily collected for transplantation. Blocking CXC-chemokine receptor type 4 (CXCR4), which binds to SDF-1, is known to dislodge leukemia stem cells (LSCs) from the BM stroma, which then become susceptible to chemotherapy.^114,115^ Since receptor blocking can potentially influence both malignant and normal cells, novel insights behind how they competitively interact with stromal cells, especially from a biophysical perspective, will be informative to devise strategies to eliminate malignant cells but to maintain normal blood cells. In addition, the ability to deliver healthy stromal cells and to subsequently control their biophysical interactions with blood cells *in vivo* can facilitate clinical translation of biophysical perturbation strategies into the treatment of cancer.

**FIG. 5.**
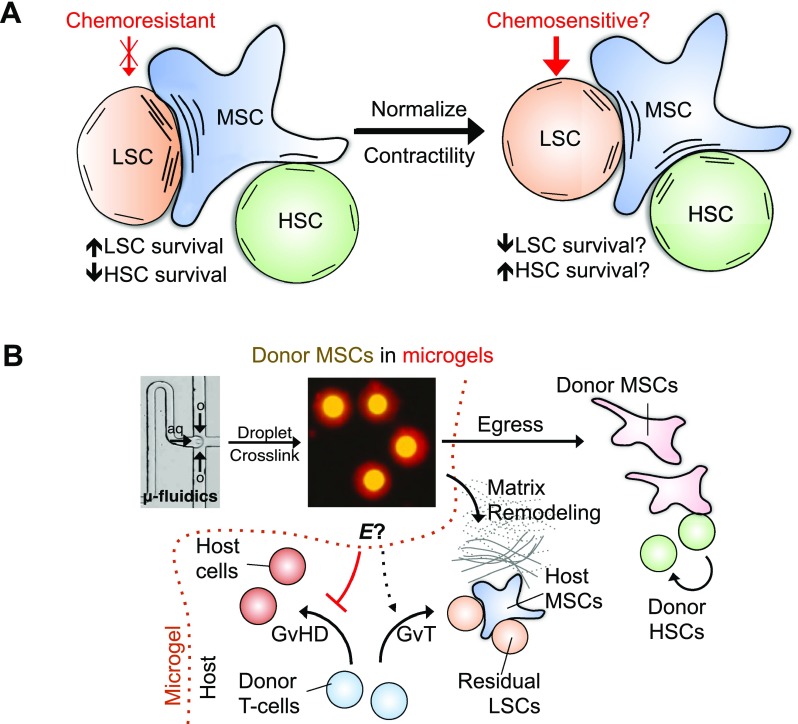
Hypothesized therapeutic strategies against blood cancers by leveraging insights into mechanobiology. (a) The binding of LSCs to MSCs leads to chemoresistance. If the interaction force between LSCs and MSCs is strong, this can polarize cortical tension on MSCs, leading to a weaker interaction between MSCs and HSCs, and hence impaired normal hematopoiesis. Normalizing cortical tension by myosin-II inhibitors may help to equalize LSC-MSC and HSC-MSC binding forces, which can potentially render LSCs chemosensitive, while maintaining HSCs. (b) After allogeneic hematopoietic transplantation in patients with primary myelofibrosis, donor MSCs (yellow) can be delivered via intrabone transplantation after encapsulation in Arg-Gly-Asp (RGD)-modified alginate microgels (red, ∼3 *μ*m thickness) using droplet microfluidics. Donor MSCs in microgels can be initially shielded from the pathological host marrow with bone deposition to prevent mechanoactivation, while secreting soluble factors to suppress GvHD. GvT may also be preserved depending on biophysical and biochemical cues from the microgels. Once the host matrix is remodeled by MMPs from donor MSCs, they can be programmed to egress from the microgels and integrate with the host to maintain donor HSCs.

Previous studies show that LSCs may be able to hijack the BM niche to decrease the engraftment of normal HSCs.^116^ While some molecular components such as CXCR4 have been implicated in this process, whether LSCs physically compete with HSCs for stromal cell influence remains unknown. While a number of cancer cell types are known to be softer than their normal counterparts,^117^ other cancer cells, especially those that show metastatic and invasive phenotypes, have been recently shown to be stiffer.^118–120^ It is important to note that as leukemia cells are exposed to chemotherapy treatment, they become more rigid by nearly 100-fold.^121^ Therefore, it can be hypothesized that chemotherapy-conditioned, rigid LSCs can polarize cortical tension on stromal cells, which can then inhibit the interaction between HSCs and stromal cells. Although blocking cell surface interactions may inhibit cell-cell adhesion and liberate both LSCs and HSCs from the BM niche, inhibiting cellular contractility is known to normalize cortical tension in cells that experience different mechanical loads while maintaining cell adhesion.^98,122^ Therefore, it will be interesting to test whether inhibitors against cellular contractility can facilitate elimination of LSCs upon chemotherapy while keeping HSCs in the BM niche [Fig. 5(a)].

The ability to encapsulate donor cells in engineered hydrogels at the single cell level has great potential in facilitating the use of biophysical cues for cancer therapy because it is possible to precisely tune the local substrate mechanics presented to individual donor cells and to control how fast the donor cells escape from the encapsulating materials to integrate with the host.^123^ For example, in leukemia patients, graft-versus-host disease (GvHD) is a major complication of allogenic hematopoietic transplantation as a result of donor T-cells recognizing foreign cells and subsequently attacking host tissue.^113^ However, donor T-cells are required to maximize the survival of patients because they can contribute to the elimination of residual cancer cells by graft-versus-tumor (GvT) effects.^124^ MSCs have been tested clinically to minimize GvHD after hematopoietic transplantation since they can suppress donor T-cell activation.^125^ However, since bone deposition remains persistent in some chronic blood cancers, such as primary myelofibrosis after transplantation,^126^ there is a risk for donor MSCs to undergo osteogenesis because of the potential mechanoactivation by the stiff interface, which can impair their immunomodulatory functions.^94^ Delivering microgels with encapsulated MSCs to the BM via intrabone injection has potential to enable the donor cells to communicate with the host initially through paracrine secretions to alleviate GvHD while remaining physically isolated from the pathological niche [Fig. 5(b)]. These microgels may be programmed to present a combination of specific biophysical or biochemical cues to encapsulated donor MSCs so that GvT effects may be preserved. Donor MSCs can also remodel the host matrix by secreting MMPs,^127^ which may help to reduce bone deposition in myelofibrosis. After matrix remodeling, MSCs can egress from the microgels and integrate with the BM so that they can contribute to the long-term maintenance of transplanted HSCs.

## IX. CONCLUSIONS

The hematopoietic system has served as an important clinical model to understand how tumor microenvironments regulate malignancy and to provide information about cancer therapy. Leveraging this system to understand cancer mechanobiology allows investigators to gain more comprehensive understanding of how biophysical cues regulate the interplay of malignant cells, hematopoietic cells, and stromal cells in cancer pathology and therapy. A wealth of information for disease causing mutations, signaling pathways, and tumor heterogeneity in the fields of hematology and oncology provides invaluable opportunities for understanding how these factors influence or are influenced by biophysical factors. Novel approaches such as using small molecule inhibitors targeting mechanosensing pathways and biomaterial strategies to modulate biophysical cues *in vivo* will facilitate the translation of the insights in cancer mechanobiology to the clinic.
